# HDVdb: A Comprehensive Hepatitis D Virus Database

**DOI:** 10.3390/v12050538

**Published:** 2020-05-14

**Authors:** Zainab Usman, Stoyan Velkov, Ulrike Protzer, Michael Roggendorf, Dmitrij Frishman, Hadi Karimzadeh

**Affiliations:** 1 Department of Bioinformatics, Wissenschaftszentrum Weihenstephan, Technische Universität München, 85354 Freising, Germany; zainabasrar@gmail.com (Z.U.); d.frishman@wzw.tum.de (D.F.); 2 Institute of Virology, Technische Universität München, 81675 Munich, Germany; stoyan.velkov@tum.de (S.V.); protzer@tum.de (U.P.); michael.roggendorf@tum.de (M.R.); 3 Division of Clinical Pharmacology, University Hospital, LMU Munich, 80337 Munich, Germany

**Keywords:** hepatitis delta virus, database, genotyping, Webserver

## Abstract

Hepatitis D virus (HDV) causes the most severe form of viral hepatitis, which may rapidly progress to liver cirrhosis and hepatocellular carcinoma (HCC). It has been estimated that 15–20 million people worldwide are suffering from the chronic HDV infection. Currently, no effective therapies are available to treat acute or chronic HDV infection. The remarkable sequence variability of the HDV genome, particularly within the hypervariable region has resulted in the provisional classification of eight major genotypes and various subtypes. We have developed a specialized database, HDVdb, which contains a collection of partial and complete HDV genomic sequences obtained from the GenBank and from our own patient cohort. HDVdb enables the researchers to investigate the genetic variability of all available HDV sequences, correlation of genotypes to epidemiology and pathogenesis. Additionally, it will contribute in understanding the drug resistant mutations and develop effective vaccines against HDV infection. The database can be accessed through a web interface that allows for static and dynamic queries and offers integrated generic and specialized sequence analysis tools, such as annotation, genotyping, primer prediction, and phylogenetic analyses.

## 1. Introduction

Hepatitis D virus (HDV) infection remains the most difficult-to-treat form of viral hepatitis, affecting 15–20 million patients worldwide with chronic hepatitis, liver cirrhosis and hepatocellular carcinoma (HCC) [1]. The HDV infection in humans occurs so far only together with hepatitis B virus (HBV) because HDV needs the envelope proteins from HBV to complete its life cycle. Therefore, two main forms of HDV infection have been described: (1) coinfection; with a high rate of viral clearance in adults similar to HBV mono-infection [2], or (2) super-infection in the presence of a pre-existing HBV infection. The latter results in a persistent chronic HDV infection in 70–90% of the cases and is associated with an early risk to develop cirrhosis and HCC [3]. The current anti-HDV therapy is mainly based on administration of interferon with a very low response rate in patients [4,5] and high chance of relapse upon discontinuation [6]. Nevertheless, efforts have been made recently to develop new anti-HDV drugs to treat chronic HDV infection, with promising results in the clinical trials [7,8,9,10,11].

HDV is a small, spherical virus of 35–37 nm in diameter, with an envelope containing the hepatitis B surface antigen (HBsAg), which surrounds the genomic RNA-nucleoprotein complex [12]. The genome is a negative sense single-stranded RNA (1.67 kb), whose complementary strand (antigenomic RNA) contains one single functional open reading frame (ORF) encoding two isoforms of the hepatitis delta antigen (HDAg), the small (*S*-HDAg, 195 aa) and the large (*L*-HDAg, 214 aa) [13,14]. The sequence encoding these isoform proteins resides in the antigenomic RNA, which, as a result of the cellular editing activity of ADAR-1, modifies the amber stop codon (UAG) to (UGG) of *S*-HDAg, resulting in the extension of the amino acid sequences by 19–20 aa at the C terminus [15].

HDV RNA sequences identified so far have been classified into eight known genotypes (HDV-1–8) based both on the nucleotide and amino acid sequences of the coding region of HDAg [16]. These genotypes are distributed across different geographical regions. In our recent studies, we identified and introduced different subtypes for the genotype 1, genotype 2 and genotype 4 [17]. Subgrouping of the so far identified HDV genotypes into distinct clusters or subtypes has been also suggested by others [18,19]. These data provide a clearer picture of the geographical a global distribution of HDV isolates. However, it is not known how these subtypes correlate with the clinical manifestation and response to therapy.

HDV-1 is the most geographically widespread genotype distributed across major regions such as Europe, Middle East, East Asia, America and Africa; whereas all other genotypes (HDV-2–8) are associated with distinct geographical and ethnic regions. HDV-2 and HDV-4 are found in North Asia and East Asia, respectively [20,21,22,23]; HDV-3 is exclusively found in the north part of South America (Brazil, Peru, Colombia, Argentina, Ecuador and Venezuela) [24,25,26,27,28] and HDV-5 to HDV-8 were previously described to be found “only” in Africa [29,30], however, a recent study reported HDV-8 isolates from Northeast Brazil, which presumably crossed the ocean through slave trades in the 16–18th centuries [31]. In humans, HDV infections with different genotypes exhibit different clinical courses and outcomes. For instance, HDV-1 strains show a broad spectrum of virulent and pathogenic phenotypes [32], HDV-2 (and HDV-4) cause milder forms of liver disease [33], whereas HDV-3 isolates are associated with outbreaks of fulminant hepatitis in South America [24]. The pathogenic properties of HDV 5–8 isolates are not well characterized [34].

For decades, HDV has been thought to have evolved in humans in combination with HBV as a helper virus providing a viral envelope to form infectious particles. Recently, however, HDV like sequences have been identified in a variety of animals and insects [35,36,37]. Sequence analysis in ducks revealed an approximately 1700-nt circular RNA genome with self-complementary, unbranched rod-like structures, and coiled-coil domains [36]. The predicted HDV-like protein discovered in ducks shares 32% amino acid similarity with the small delta antigen (*S*-HDAg) of the human HDV (hHDV).

This discovery of an HDV-like agent in ducks was followed by the identification of a deltavirus in snakes (*Boa constrictor*), designated as snake HDV (sHDV) [37]. Sequence comparison of the snake delta antigen (sHDAg) showed that its aa sequence is 55% identical to its human counterparts. Anti-sera raised against a recombinant sHDAg was used in immunohistology studies. A broad viral target was demonstrated in different snake cells, including neurons, epithelial cells and leukocytes. The duck and snake viruses constitute divergent phylogenetic lineages as compared to the human HDV (hHDV), which so far seem quite distant related to the known human isolates.

Using additional meta-transcriptomic data, highly divergent HDV-like viruses were also found to be present in fish, amphibians and invertebrates. These newly identified viruses share human HDV-like genomic features such as a small genome size of 1.7 kb in length [35].

The identification of a much broader range of hosts as initially anticipated and the fact that the HDV RNA genome can efficiently replicate in different tissues and species, raise the possibility that HDV is able to be transmitted independently of HBV. Perez-Vargas J. et al. [38] have shown, that envelope glycoproteins (GPs) of unrelated viruses can act as helper viruses for HDV including vesiculovirus, flavivirus and hepacivirus. These GPs can package HDV RNPs, allowing efficient egress of HDV particles in the extracellular milieu of coinfected cells and subsequent entry into the cells expressing the corresponding receptors. In vivo studies in humanized mice indicate that HDV RNPs packaged into an HCV envelope can propagate HDV infection in the liver of coinfected mice [38].

In recent years, the amount of HDV genomic data has increased exponentially. Intensive sequencing efforts have resulted in approximately 2621 nucleotide HDV sequences (partial and full length) deposited into the DDBJ, EMBL and GenBank databases. The GenBank is part of the International Nucleotide Sequence Database Collaboration (INSDC), which comprises of the DNA DataBank of Japan (DDBJ), the European Nucleotide Archive (ENA) and GenBank at NCBI. Those three organizations are synchronized and exchange data on a daily basis. Therefore, the sequences dataset can be retrieved using any of the platforms, i.e., the GenBank database. In order to exploit this large and growing collection of sequences efficiently and to facilitate sequence analysis we sought to develop a specialized database. Databases established for other types of viruses, in particular for HIV [39], HBV [40] and HCV [41] have proved to be very helpful for epidemiological and clinical studies, more importantly in characterizing resistance to direct anti-viral drugs. Here, we present the hepatitis delta virus database (HDVdb; http://hdvdb.bio.wzw.tum.de/). This comprehensive database collates HDV sequences and is mainly oriented towards the sequence analysis of HDV isolates, including the complete viral genomic sequences, large and small HDV antigen sequences (*L*-HDAg and *S*-HDAg, respectively). HDVdb provides a platform for genotyping and phylogenetic analyses including prediction of HDV genotypes for user-supplied HDV sequence entries. Moreover, the database will help in identifying the emerging variants related to immune escape from the B and T cell response as described recently [42,43] and in detecting therapy resistant variants across different HDV genotypes, which can be correlated with clinical studies.

## 2. Materials and Methods

The HDVdb building process began with the manual retrieval of all the HDV entries using the keyword: “hepatitis delta virus” from GenBank hosted at NCBI [44]. All results corresponding to taxon “Hepatitis delta virus” (taxid: 12475) were considered. Currently, a total of 2621 hepatitis delta virus nucleotide sequences are deposited into GenBank. These entries contain full sequence records of both HDV “complete genomic” sequences and subgenomic fragments (*S*-HDAg; 1–195 aa and *L*-HDAg; 1–214 aa) as well as partial cds sequences. GenBank entries containing complete HDV protein sequences were also incorporated. Majority of the sequences were retained to provide the maximum data information to our visitors, however, sequences shorter than 90 bases were not included into the dataset. The sequence dataset was than parsed by creating an automatic pipeline using Java programming language to extract essential information for each accession number such as strain name, genotype, country and date. In addition, 152 sequences lacking the genotype information were assigned to a genotype by performing similarity search using BLAST [45].

**Figure 1:**
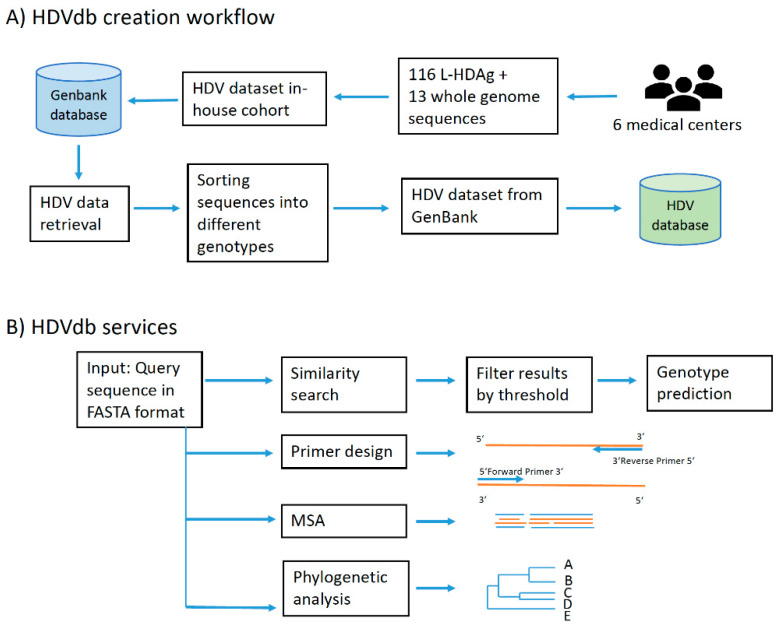
HDVdb construction and analysis workflow. (**A**) Building blocks of HDVdb based on publicly available and in-house isolates. (**B**) List of services available at the HDVdb. In Primer design, the orange lines represent template and blue lines represent the primers. In MSA (Multiple sequence alignment): blue and orange lines represent different aligned sequences, schematically.

The HDVdb web interface is hosted using Apache HTTP server and runs on PHP Laravel framework. The HDVdb is updated on an annual basis. The software for the automatic annotation, as well as for the querying and the managing the database is implemented in Java and Bash programming languages. It makes use of all defined genotypes and their subgenotypes. The workflow of database construction is schematically demonstrated in Figure 1.

## 3. Results and Discussion

**Figure 2:**
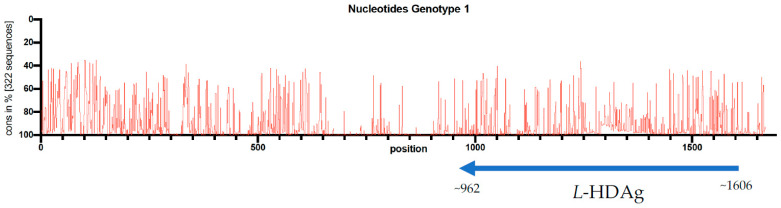
Conservation rate of 322 full genome nucleotide sequences of the HDV genotype 1. The single open reading frame of HDAg is located on antigenomic strand between position 962 and 1606 and indicated with an arrow under the genome.

The HDVdb is accessible online through the website: http://hdvdb.bio.wzw.tum.de/. HDVdb contains entries for human hepatitis delta virus sequences, with 512 complete genome sequences, as well as 1066 *L*-HDAg and *S*-HDAg and 1281 partial cds nucleotide sequences as well as protein sequences for *L*-HDAg and *S*-HDAg. These sequences can be directly downloaded from the database for any further analysis. Links to protein sequences for both *L*-HDAg and *S*-HDAg sequences are directly provided at the home page. Additionally, we included 13 complete genome (Accession MH457142-MH457154) and 116 *L*-HDAg sequences (Accession MF175257-MF175360, MH447633-MH447644) retrieved from six different medical centers of our European study cohort [17]. In this study, sequence conservation at each position across the entire length of the 322 multiply aligned genome sequences (i.e., genotype-1) was visualized. The multiple sequence alignments were performed using MUSCLE v3.8.5551 [46] whereas the evaluation was performed using customized Ruby scripts (Figure 2). We concluded that despite low conservation rate throughout the HDV genome, there were no significant differences on genotyping results using the whole genome or the *L*-HDAg encoding region.

**Figure 3:**
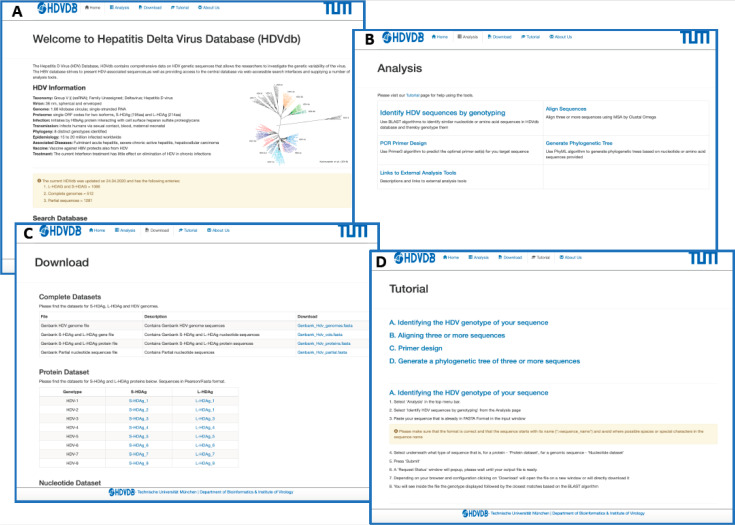
Web interface of the HDVdb. (**A**) The homepage, with the menu bar and all the menus repeated as buttons for ease-of-use. The page summarizes the characteristics and statistics of HDV. The nucleotide as well as protein datasets can be directly accessed through the homepage. The sequence files can be viewed for each genotype both in protein and nucleotide. (**B**) List of tools available on the website to analyze HDV related sequences. (**C**) List of sequence datasets available in FASTA format and updated on a regular basis. (**D**) A snapshot of the tutorial page with step-by-step instructions on how to use different tools available on this database.

The HDVdb is divided into a static and a dynamic part as demonstrated in Figure 3. The static part allows the user to access the general information about HDV. The homepage provides a data summary of updated number of *S*-HDAg, *L*-HDAg and complete genomes of all the eight known genotypes on the database. The user can retrieve pre-compiled protein and nucleotide datasets for complete genome, *L*-HDAg, and *S*-HDAg separately for each genotype, alternatively the user can also download a single FASTA file containing these datasets for all genotypes. In addition, the database also provides a tutorial to help the users with necessary technical information required to access tools available on the database.

The dynamic part allows the analysis of user-provided queries. The homepage presents an interactive search box that allows the visitors of our database with options to search sequences based on accession number, genotype, country and date for protein, complete genome, coding sequences for *L*-HDAg and *S*-HDAg, as well as partial sequences. The nucleotide and protein sequences queries can be genotyped using “Identify HDV sequences by genotyping” option. The webservice uses BLAST [45], which performs local alignments and scores the most relevant sequences to access the query genotype. A minimum of 75% identity score against the database is required to classify the query sequence to one of the HDV genotypes. This threshold prevents the false positives to be classified and is based on our previous research [17].

Furthermore, we integrated computational tools for multiple sequence analysis (Clustal Omega, version 1.2.3 [47]), primer design (Primer3, version 2.3.7 [48]) and phylogenetic analyses (Phylip (PhyML), version 3.696 [49]), Figure 3. The user can also graphically visualize the phylogenetic trees on completion generated by FigTree, version 1.4.4. The request and response from these services was handled using PHP Laravel framework and bash scripting.

## 4. Conclusions

Hepatitis D has received a lot of attention in recent years, resulting in a flood of new findings and information, including next generation sequencing data. However, a platform capable of collecting and analyzing this growing body of data has so far been missing. Here we introduced HDVdb as a comprehensive database of human HDV sequences with a potential of expansion to the recently identified isolates from animals and insects. HDVdb allows the user to download structured data of all known HDV sequences. It also permits the user to use this data and perform comparative sequence analysis using multiple bioinformatics services available directly on HDVdb website.

## References

[1] Chen H.Y., Shen D.T., Ji D.Z., Han P.C., Zhang W.M., Ma J.F., Chen W.S., Goyal H., Pan S., Xu H.G. (2018). Prevalence and burden of hepatitis D virus infection in the global population: A systematic review and meta-analysis. Gut.

[2] Noureddin M., Gish R. (2014). Hepatitis delta: Epidemiology, diagnosis and management 36 years after discovery. Curr. Gastroenterol. Rep..

[3] Smedile A., Farci P., Verme G., Caredda F., Cargnel A., Caporaso N., Dentico P., Trepo C., Opolon P., Gimson A. (1982). Influence of delta infection on severity of hepatitis B. Lancet.

[4] Wedemeyer H., Yurdaydìn C., Dalekos G.N., Erhardt A., Çakaloğlu Y., Değertekin H., Gürel S., Zeuzem S., Zachou K., Bozkaya H. (2011). Peginterferon plus adefovir versus either drug alone for hepatitis delta. N. Engl. J. Med..

[5] Wedemeyer H., Yurdaydin C., Hardtke S., Caruntu F.A., Curescu M.G., Yalcin K., Akarca U.S., Gurel S., Zeuzem S., Erhardt A. (2019). Peginterferon alfa-2a plus tenofovir disoproxil fumarate for hepatitis D (HIDIT-II): A randomised, placebo controlled, phase 2 trial. Lancet Infect. Dis..

[6] Heidrich B., Yurdaydin C., Kabacam G., Ratsch B.A., Zachou K., Bremer B., Dalekos G.N., Erhardt A., Tabak F., Yalcin K. (2014). Late HDV RNA relapse after peginterferon alpha-based therapy of chronic hepatitis delta. Hepatology.

[7] Bogomolov P., Alexandrov A., Voronkova N., Macievich M., Kokina K., Petrachenkova M., Lehr T., Lempp F.A., Wedemeyer H., Haag M. (2016). Treatment of chronic hepatitis D with the entry inhibitor myrcludex B: First results of a phase Ib/IIa study. J. Hepatol..

[8] Beilstein F., Blanchet M., Vaillant A., Sureau C. (2018). Nucleic acid polymers are active against hepatitis delta virus infection in vitro. J. Virol..

[9] Zhang Z., Filzmayer C., Ni Y., Sultmann H., Mutz P., Hiet M.S., Vondran F.W.R., Bartenschlager R., Urban S. (2018). Hepatitis D virus replication is sensed by MDA5 and induces IFN-beta/lambda responses in hepatocytes. J. Hepatol..

[10] Bazinet M., Pantea V., Cebotarescu V., Cojuhari L., Jimbei P., Albrecht J., Schmid P., Le Gal F., Gordien E., Krawczyk A. (2017). Safety and efficacy of REP 2139 and pegylated interferon alfa-2a for treatment-naive patients with chronic hepatitis B virus and hepatitis D virus co-infection (REP 301 and REP 301-LTF): A non-randomised, open-label, phase 2 trial. Lancet Gastroenterol. Hepatol..

[11] Lempp F.A., Urban S. (2017). Hepatitis delta virus: Replication strategy and upcoming therapeutic options for a neglected human pathogen. Viruses.

[12] Lai M.M. (1995). The molecular biology of hepatitis delta virus. Annu. Rev. Biochem..

[13] Huang C.R., Lo S.J. (2010). Evolution and diversity of the human hepatitis d virus genome. Adv. Bioinform..

[14] Weiner A.J., Choo Q.L., Wang K.S., Govindarajan S., Redeker A.G., Gerin J.L., Houghton M. (1988). A single antigenomic open reading frame of the hepatitis delta virus encodes the epitope(s) of both hepatitis delta antigen polypeptides p24 delta and p27 delta. J. Virol..

[15] Casey J.L. (2006). RNA editing in hepatitis delta virus. Curr. Top. Microbiol. Immunol..

[16] Le Gal F., Gault E., Ripault M.P., Serpaggi J., Trinchet J.C., Gordien E., Deny P. (2006). Eighth major clade for hepatitis delta virus. Emerg. Infect. Dis..

[17] Karimzadeh H., Usman Z., Frishman D., Roggendorf M. (2019). Genetic diversity of hepatitis D virus genotype-1 in Europe allows classification into subtypes. J. Viral Hepat..

[18] Le Gal F., Brichler S., Drugan T., Alloui C., Roulot D., Pawlotsky J.M., Deny P., Gordien E. (2017). Genetic diversity and worldwide distribution of the deltavirus genus: A study of 2,152 clinical strains. Hepatology.

[19] Paul Dény C.D., Gatherer D., Kay A.C., Le Gal F., Drugan T., Mario Rizzetto A.S. (2019). Hepatitis delta virus clades and genotypes: A practical approach. Hepatitis D. Virology, Management and Methodology.

[20] Ivaniushina V., Radjef N., Alexeeva M., Gault E., Semenov S., Salhi M., Kiselev O., Deny P. (2001). Hepatitis delta virus genotypes I and II cocirculate in an endemic area of Yakutia, Russia. J. Gen. Virol..

[21] Lee C.M., Changchien C.S., Chung J.C., Liaw Y.F. (1996). Characterization of a new genotype II hepatitis delta virus from Taiwan. J. Med. Virol..

[22] Sakugawa H., Nakasone H., Nakayoshi T., Kawakami Y., Miyazato S., Kinjo F., Saito A., Ma S.P., Hotta H., Kinoshita M. (1999). Hepatitis delta virus genotype IIb predominates in an endemic area, Okinawa, Japan. J. Med. Virol..

[23] Marino Q., Cisse M., Gerber A., Dolo O., Sayon S., Ba A., Brichler S., Tata Traore F., Gordien E., Togo J. (2019). Low hepatitis D seroprevalence in blood donors of Bamako, Mali. Infect. Dis. (Lond.).

[24] Casey J.L., Brown T.L., Colan E.J., Wignall F.S., Gerin J.L. (1993). A genotype of hepatitis D virus that occurs in northern South America. Proc. Natl. Acad. Sci. USA.

[25] Gomes-Gouvea M.S., Soares M.C., Bensabath G., de Carvalho-Mello I.M., Brito E.M., Souza O.S., Queiroz A.T., Carrilho F.J., Pinho J.R. (2009). Hepatitis B virus and hepatitis delta virus genotypes in outbreaks of fulminant hepatitis (Labrea black fever) in the western Brazilian Amazon region. J. Gen. Virol..

[26] Quintero A., Uzcategui N., Loureiro C.L., Villegas L., Illarramendi X., Guevara M.E., Ludert J.E., Blitz L., Liprandi F., Pujol F.H. (2001). Hepatitis delta virus genotypes I and III circulate associated with hepatitis B virus genotype F in Venezuela. J. Med. Virol..

[27] Scarponi C.F.O., Silva R.D.N.D., Souza Filho J.A., Guerra M.R.L., Pedrosa M.A.F., Mol M.P.G. (2019). Hepatitis delta prevalence in South America: A systematic review and meta-analysis. Rev. Soc. Bras. Med. Trop..

[28] di Filippo Villa D., Cortes-Mancera F., Payares E., Montes N., de la Hoz F., Arbelaez M.P., Correa G., Navas M.C. (2015). Hepatitis D virus and hepatitis B virus infection in Amerindian communities of the Amazonas state, Colombia. Virol. J..

[29] Makuwa M., Caron M., Souquiere S., Malonga-Mouelet G., Mahe A., Kazanji M. (2008). Prevalence and genetic diversity of hepatitis B and delta viruses in pregnant women in Gabon: Molecular evidence that hepatitis delta virus clade 8 originates from and is endemic in central Africa. J. Clin. Microbiol..

[30] Radjef N., Gordien E., Ivaniushina V., Gault E., Anais P., Drugan T., Trinchet J.C., Roulot D., Tamby M., Milinkovitch M.C. (2004). Molecular phylogenetic analyses indicate a wide and ancient radiation of African hepatitis delta virus, suggesting a deltavirus genus of at least seven major clades. J. Virol..

[31] Santos M.D., Gomes-Gouvêa M.S., Nunes J.D., Barros L.M., Carrilho F.J., Ferreira A.e.S., Pinho J.R. (2016). The hepatitis delta genotype 8 in Northeast Brazil: The North Atlantic slave trade as the potential route for infection. Virus Res..

[32] Niro G.A., Smedile A., Andriulli A., Rizzetto M., Gerin J.L., Casey J.L. (1997). The predominance of hepatitis delta virus genotype I among chronically infected Italian patients. Hepatology.

[33] Su C.W., Huang Y.H., Huo T.I., Shih H.H., Sheen I.J., Chen S.W., Lee P.C., Lee S.D., Wu J.C. (2006). Genotypes and viremia of hepatitis B and D viruses are associated with outcomes of chronic hepatitis D patients. Gastroenterology.

[34] Barros L.M., Gomes-Gouvea M.S., Pinho J.R., Alvarado-Mora M.V., Dos Santos A., Mendes-Correa M.C., Caldas A.J., Sousa M.T., Santos M.D., Ferreira A.S. (2011). Hepatitis Delta virus genotype 8 infection in Northeast Brazil: Inheritance from African slaves?. Virus Res..

[35] Chang W.S., Pettersson J.H., Le Lay C., Shi M., Lo N., Wille M., Eden J.S., Holmes E.C. (2019). Novel hepatitis D-like agents in vertebrates and invertebrates. Virus Evol..

[36] Wille M., Netter H.J., Littlejohn M., Yuen L., Shi M., Eden J.S., Klaassen M., Holmes E.C., Hurt A.C. (2018). A divergent hepatitis D-like agent in birds. Viruses.

[37] Hetzel U., Szirovicza L., Smura T., Prahauser B., Vapalahti O., Kipar A., Hepojoki J. (2019). Identification of a novel deltavirus in boa constrictors. mBio.

[38] Perez-Vargas J., Amirache F., Boson B., Mialon C., Freitas N., Sureau C., Fusil F., Cosset F.L. (2019). Enveloped viruses distinct from HBV induce dissemination of hepatitis D virus in vivo. Nat. Commun..

[39] Kuiken C., Korber B., Shafer R.W. (2003). HIV sequence databases. Aids Rev..

[40] Hayer J., Jadeau F., Deleage G., Kay A., Zoulim F., Combet C. (2013). HBVdb: A knowledge database for Hepatitis B Virus. Nucleic Acids Res..

[41] Kuiken C., Hraber P., Thurmond J., Yusim K. (2008). The hepatitis C sequence database in Los Alamos. Nucleic Acids Res..

[42] Kefalakes H., Koh C., Sidney J., Amanakis G., Sette A., Heller T., Rehermann B. (2019). Hepatitis D Virus-specific CD8(+) T cells have a memory-like phenotype associated with viral immune escape in patients with chronic hepatitis D virus infection. Gastroenterology.

[43] Karimzadeh H., Kiraithe M.M., Oberhardt V., Salimi Alizei E., Bockmann J., Schulze Zur Wiesch J., Budeus B., Hoffmann D., Wedemeyer H., Cornberg M. (2019). Mutations in hepatitis D virus allow it to escape detection by CD8(+) T cells and evolve at the population level. Gastroenterology.

[44] Benson D.A., Clark K., Karsch-Mizrachi I., Lipman D.J., Ostell J., Sayers E.W. (2015). GenBank. Nucleic Acids Res..

[45] Altschul S.F., Gish W., Miller W., Myers E.W., Lipman D.J. (1990). Basic local alignment search tool. J. Mol. Biol..

[46] Edgar R.C. (2004). MUSCLE: Multiple sequence alignment with high accuracy and high throughput. Nucleic Acids Res..

[47] Sievers F., Wilm A., Dineen D., Gibson T.J., Karplus K., Li W., Lopez R., McWilliam H., Remmert M., Soding J. (2011). Fast, scalable generation of high-quality protein multiple sequence alignments using Clustal Omega. Mol. Syst. Biol..

[48] Untergasser A., Cutcutache I., Koressaar T., Ye J., Faircloth B.C., Remm M., Rozen S.G. (2012). Primer3—New capabilities and interfaces. Nucleic Acids Res..

[49] Guindon S., Dufayard J.F., Lefort V., Anisimova M., Hordijk W., Gascuel O. (2010). New algorithms and methods to estimate maximum-likelihood phylogenies: Assessing the performance of PhyML 3.0. Syst. Biol..

